# Villainous role of estrogen in macrophage-nerve interaction in endometriosis

**DOI:** 10.1186/s12958-018-0441-z

**Published:** 2018-12-05

**Authors:** Yanchun Liang, Hongyu Xie, Jinjie Wu, Duo Liu, Shuzhong Yao

**Affiliations:** 1 Department of Obstetrics and Gynecology, First Affiliated Hospital, Sun Yat-sen University, No. 58, the 2nd Zhongshan Road, Yuexiu District, Guangzhou, Guangdong Province China; 2 Grade 2012 in 8-year Medical Education Program of Zhongshan School of Medicine, Sun Yat-sen University, Guangzhou, 510089 China

**Keywords:** Estrogen, Macrophage, Nerve fiber, Neuroinflammation, Endometriosis

## Abstract

Endometriosis is a complex and heterogeneous disorder with unknown etiology. Dysregulation of macrophages and innervation are important factors influencing the pathogenesis of endometriosis-associated pain. It is known to be an estrogen-dependent disease, estrogen can promote secretion of chemokines from peripheral nerves, enhancing the recruitment and polarization of macrophages in endometriotic tissue. Macrophages have a role in the expression of multiple nerve growth factors (NGF), which mediates the imbalance of neurogenesis in an estrogen-dependent manner. Under the influence of estrogen, co-existence of macrophages and nerves induces an innovative neuro-immune communication. Persistent stimulation by inflammatory cytokines from macrophages on nociceptors of peripheral nerves aggravates neuroinflammation through the release of inflammatory neurotransmitters. This neuro-immune interaction regulated by estrogen sensitizes peripheral nerves, leading to neuropathic pain in endometriosis. The aim of this review is to highlight the significance of estrogen in the interaction between macrophages and nerve fibers, and to suggest a potentially valuable therapeutic target for endometriosis-associated pain.

## Introduction

Endometriosis is a common gynecological disorder, which is defined as the presence of the endometrial-like tissue outside the uterine cavity [1]. This chronic inflammatory disease affects 6–10% of women during reproductive age [2], and 30 to 50% are accompanied with pelvic pain and is associated with infertility [3, 4]. Though extensive research has proposed many theories for the pathogenesis of endometriosis, the mechanism of endometriosis-associated pain still remains elusive. As an estrogen-dependent disease, local levels of estrogen in the menstrual blood and peritoneal fluids are higher in patients with endometriosis in comparison to healthy women [5]. Estrogen exhibits a pivotal role in the maintenance of endometriosis-associated pain.

Growing evidences support that the presence of endometriosis-associated nerve fibers is related to the severity of dysmenorrhea [6]. Studies have confirmed the presence and high expression of sensory, sympathetic and parasympathetic nerve fibers in peritoneal lesion in comparison to the normal peritoneum [7]. In addition to the aberrant distribution of nerves, impairment of immune microenvironment in the peritoneal cavity induces inflammation which can also mediate endometriosis-associated pain [8]. Endometriosis-associated inflammation is able to stimulate and sensitize peripheral nerves. Researchers found that activation of (DRG) mast cells by estrogen can trigger the release of NGF and also sensitize dorsal root ganglion cells [9]. This neuroimmune interaction mediated by estrogen is correlated with the severity of endometriosis-associated dysmenorrhea. Among the dysregulated immune components, macrophages are one of the most numerous immune cells in endometriotic lesions [10]. The concentration and proportion of macrophage are significantly increased in peritoneal fluid of women with endometriosis [11, 12], indicating their involvement in the development of pain. A recent study exhibits the critical role of estrogen in mediating the communication between macrophage and nerve fibers in endometriosis [13]. However, as an estrogen-dependent disease, the exact mechanism of estrogen regulating the cross talk between macrophages and nerve fibers in endometriosis is ambiguous.

The aim of this review is to highlight the potential role of estrogen in maintaining the cross talk between macrophages and nerve fibers in endometriosis. Theoretical elaboration of the underlying mechanism provides a new insight into the pathogenesis of endometriosis and a potential therapeutic targets for treatment.

## The role of estrogen and their receptors in endometriosis

Estrogen has a central role in endometriosis. It can facilitate disease progression mainly through binding different estrogen receptors (ER) [14]. The ERs can be classically divided into two subtypes: ERα and ERβ, both act as nuclear transcription factors [15]. ERα is mainly responsible for the modulation of cell growth related genes [16], whereas ERβ plays an important role in cell cycle progression and apoptosis. Further studies reveal another subtype of estrogen receptor, membrane-association G-protein coupled estrogen receptor (GPER) in the cell menmbrane, which is able to mediate the rapid non-genomic effects of estrogen [17]. GPER is reported to be capable of activating several downstream molecules, such as ERK1/2, PI3K, PKC and calcium ion channels [17]. These studies suggest that different signaling pathways are activated by different kinds of ER, exhibiting their distinct function. As a result, the action of estrogen on endometriosis is closely related to the types of receptors and their distribution.

### Distribution of estrogen receptors in endometriosis

Different estrogen receptors have abnormal distribution in the ectopic endometrium of women with endometriosis [18]. A higher ERβ and a lower ERα expression profile in endometriotic lesions have been proposed as a major background of estrogen action in endometriosis [19]. Several studies have shown significantly elevated ERβ levels and decreased ERα levels in endometriotic stromal cells and tissues compared to the eutopic endometrium [20]. ERβ-induced the formation of ASK-1/STRAP complex can prevent the activation of tumor necrosis factor α (TNFα)-mediated apoptosis in endometriotic tissues [21]. Increased expression of Ras-like and estrogen-regulated growth inhibitor (RERG) has also been identified after the activation of ERβ [22]. ERβ binds to the promoter regulatory region of RERG, inducing gene expression of RERG, and then enhancing the proliferation of endometriotic cells. Moreover, multiple molecules in endometriosis are able to stimulate the expression of ERβ, such as insulin-like growth factors 1 (IGF1) [23], activated platelets [24], serum and glucocorticoid-regulated kinase (SGK1) [25]. However, cross-talk between ERα and interleukin (IL) 6 pathways is recently shown to promote the early initiation of endometriosis [26]. IL-6 mediates the recruitment of monocytes which can differentiate into macrophages expressing ERα. Estrogen-ERα binding in turn regulates the IL-6 promoter through activation of NF-κB and CEBPβ. Further study characterizes the heterogeneous expression of ERs in different types of endometriosis. Ovarian lesions show the lowest expression of ERα and the highest expression of ERβ, whereas the fallopian tube lesions show a high expression of both receptors [27]. The largest ERβ to ERα ratio is observed in ovarian lesions compared with peritoneal, fallopian tube, and extra pelvic lesions. Moreover, aberrant expression of ERα and ERβ are both correlated to the production of proinflammatory cytokines in endometriosis [28]. The combinational interaction of ERβ with caspase 1 and NLR family pyrin domain-containing 3 (NALP3) activates caspase 1 and subsequently elevates the level of IL-1β in ectopic lesions, enhancing the inflammation in the endometriotic microenvironment [21]. In addition, the expression of GPER is also significantly increased in ectopic endometrium compared to eutopic endometrium [29], which specifically promotes the proliferation of endometrial fragment. Although abnormal expression of different ERs has been identified in endometriotic lesion, their functions involved in the pathogenesis of endometriosis are far from clarified.

### Characteristics of estrogen receptors in macrophages of endometriosis

As discussed above, activation of the estrogen receptors can induce inflammatory events. In the endometriotic microenvironment, multiple immune cells, such as macrophages, mast cells, neutrophils, and mature dendritic cells are mobilized by the retrograded endometrial cells. Among them, macrophages are the primary contributor of pro-inflammatory cytokines and chemokines [30]. Both ERα and ERβ are significantly overexpressed in macrophages of endometriosis [28]. Treatment with 17β-estradiol in human macrophages in vitro induces an increase of ERα expression in macrophages. Furthermore, the estrogen-ER interaction can up-regulate the production of IL-6 and TNFα from lipopolysaccharide (LPS)-activated macrophages [31]. Specifically, macrophages with overexpression of ERβ in endometriotic lesions have been shown to exhibit enhanced phagocytic activity, and secrete inflammatory cytokines including IL1β, TNFα and IL6 [21, 32]. Deficiency of ERα, but not ERβ, increased TNFα production by mouse peritoneal macrophages in response to bacterial stimuli, indicating a prominent role of ERα in mediating the anti-inflammatory effect of estrogen. However, an opposite finding shows the ability of selective ERβ activation to inhibit the expression of inflammatory mediators. Some researchers even argue that ERα is only detected in a subset of macrophages in both peritoneal cavity and endometriotic biopsy specimens, which is different from previous studies [13]. Activation of ERα by estrogen is able to inhibit NF-κB-dependent inflammation by promoting synthesis of the negative regulator IκBα [33]. And this discrepancy about the ERs on macrophages is due to the inherent differences between peritoneal fluid macrophages and tissue macrophages. Moreover, the expression of GPER on macrophage in endometriosis is also significantly increased compared with that of healthy tissue, suggesting an alternative way that the immune response induced by macrophages is modulated by estrogen [34]. Treatment with the selective GPER agonist G-1 is able to inhibit LPS-induced TNFα production in human macrophages. Activation of GPER down-regulates NF-κB promoter activation through increase phosphorylation of JNK, resulting in the inhibition of LPS-induced IL-6 secretion [35]. GPER can also affects function macrophages by decreasing the expression of TLR4. These data indicate that the pro- or anti-inflammatory role of estrogen on macrophages is closely related to different types of estrogen receptors. Activation of ERβ has the potential to arouse pro-inflammatory response, while both ERα and GPER play significant roles in anti-inflammation.

### Estrogen receptors in the nervous system

In addition to macrophage, ERs are also expressed on the nervous system. Whether in the myometrium of women with or without adenomyosis, the expression of ERβ in sympathetic nerves is much more than sensory nerves [36, 37]. Activation of ERβ in sympathetic nerve could promote the down-regulation of cyclin E, IGF1, or cytokines [38], leading to a prominent reduction of the sympathetic innervation. ERα and ERβ are also expressed in the uterine-related dorsal root ganglion (DRG) neurons [39, 40], suggesting an important plasticity regulation and developmental function in uterine sensory nerves. Although ERα and ERβ are detected in DRG, the impacts of ER agonists on neuron in endometriosis have been clarified to depend on the subtypes of ERs. Activation of ERβ in DRG can down-regulate the expression of nerve repellent factor, SLIT3. Conversely, activation of ERα upregulates SLIT3 expression in DRGs, highlighting the potentially opposite impacts of different ERs in neuron [41]. Acute activation of membrane-associated ERα in peripheral sensory neurons by estrogen is confirmed to enhance allodynia elicited by the inflammatory mediator bradykinin [42]. Moreover, GPER is also identified in peripheral sensory neurons and the expression of GPER is increased after peripheral nerve injury [43]. However, the exact mechanism of GPER in neurogenesis and peripheral sensitization remains unclear.

## Estrogen mediates the interaction of macrophages and nerve fibers

As different estrogen receptors are both expressed on the macrophages and nerve fibers, estrogen is believed to be able to mediate the activity of macrophages and the characteristics of nerve fibers. Therefore, estrogen is critical to regulate the interaction between macrophages and nerve fibers. This includes the recruitment of macrophages, aberrant neurogenesis as well as the abnormal inflammation in endometriosis [13].

### Recruitment of macrophages by nerve fibers in an estrogen-dependent manner

There have been multiple studies demonstrating a significantly high level of macrophages in endometriotic milieu [44, 45]. Among different mechanisms involved in the recruitment of macrophages, nerve fibers exhibit an extraordinary ability to recruit macrophages to the site of lesion. Many factors have been confirmed to mediate the recruitment of macrophages toward the nerve fibers, including leukemia inhibitory factor (LIF) [46], IL1α, IL1β [47] and pancreatitis- associated protein 3 (PAP3) [48]. The role of estrogen during the process of nerve fiber-induced macrophage recruitment has been recently clarified [13]. Following peripheral axon injury, the neuron-derived exosomal miR-21-5p is phagocytosed by macrophages, which can mediate the macrophages infiltrations towards peripheral nerves [49]. Analysis has proven that exosomal miR-21-5p release is mediated by the estrogen signaling pathway [50]. Estrogen can also stimulate the secretion of colony-stimulating factor 1 (CSF1) and C-C motif ligand 2 (CCL2) from peripheral nerves, which enhances macrophages migration towards the endometriotic lesion [13].

**Fig. 1:**
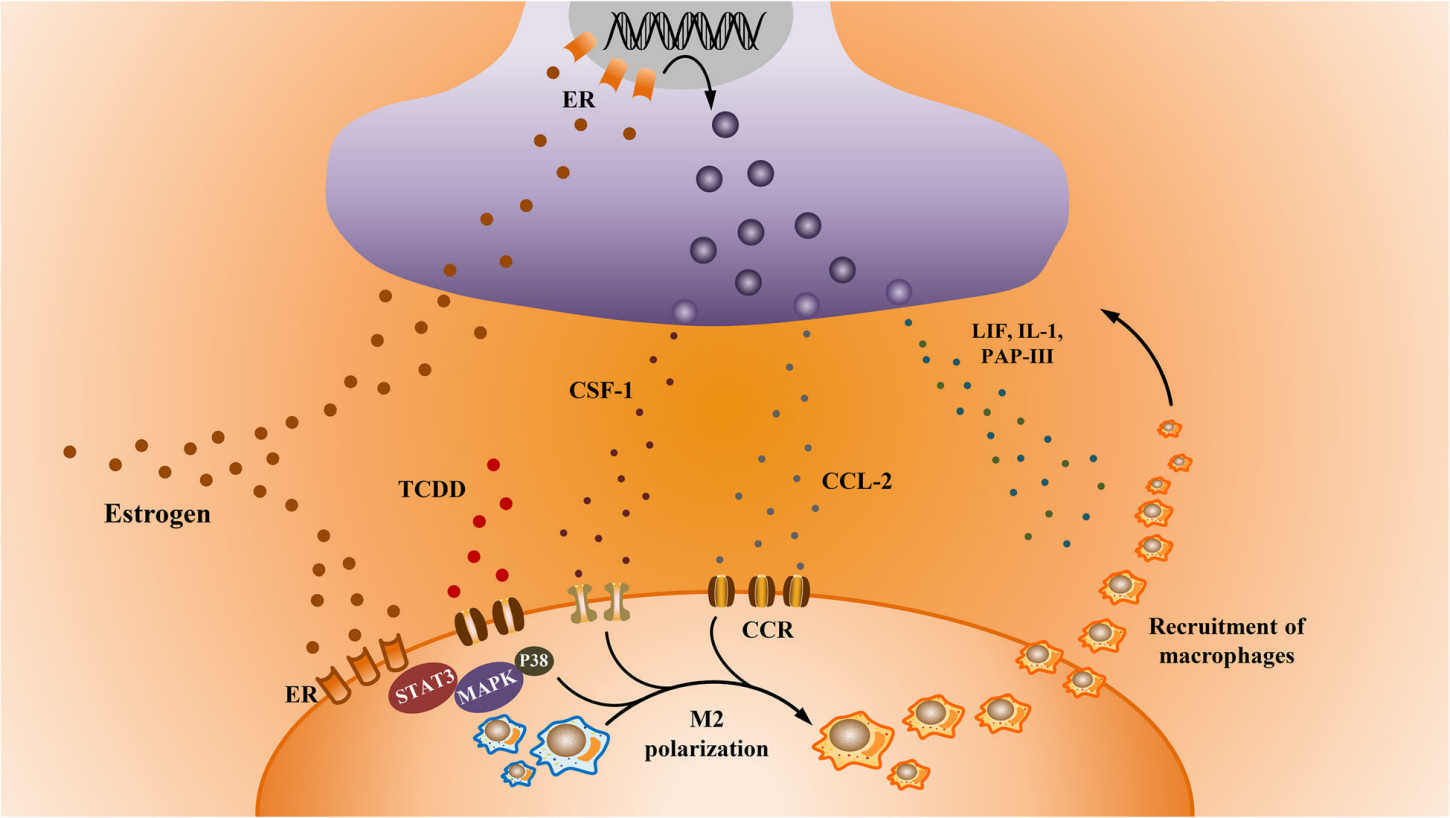
Recruitment of macrophages by nerve fibers in an estrogen-dependent manner. Upon the influence of estrogen, nerve fibers can secrete LIF, IL1 and PAP3, mediating the recruitment of macrophages. Estrogen can enhance the release of CSF1 and CCL2 to promote migration of macrophages toward peripheral nerves. The interaction of CCL2 and its receptors activates the polarization of macrophages. Synergistic effect of estrogen and TCDD can also induce the M2 polarization of macrophages through activation of the STAT3 and P38-MAPK pathway. LIF: leukemia inhibitory factor; IL1: interleukin 1; PAP3: pancreatitis- associated protein 3; CSF1: colony-stimulating factor 1; CCL2: C-C motif ligand 2; CCR: C-C motif chemokine receptor; TCDD: 2, 3, 7, 8-tetrachlorodibenzo-p-dioxin; ER: estrogen receptor

Macrophages are special immune cells with high plasticity and different functional profiles. These immune cells can be divided into two subtypes as classically activated (M1) and alternatively activated (M2) macrophages based on the distinct pathways of activation [51]. The function of M1 macrophages is to produce pro-inflammatory cytokines which is induced by Th1 cytokines, such as Interferon γ (IFNγ) and TNFα. M2 macrophages, which are anti-inflammatory, are polarized by Th2 cytokines, such as IL-4 and IL-13 via activation of STAT6 through IL-4 receptor alpha [52]. Interestingly, the macrophages in endometriosis are predominantly of M2 phenotype [53]. Further evidence confirms that abnormal endometriotic milieu can induce the polarization of M2 macrophages [54]. Estrogen-dependent regulation of CCL2 in peritoneal cavity is confirmed to polarize macrophages from M1 toward M2 phenotype via CCL2/CCR pathway [55]. 17β-estradiol and TCDD (2, 3, 7, 8-tetrachlorodibenzo-p-dioxin) also have a synergistic effect on inducing M2 macrophages polarization through activation of the STAT3 and P38-MAPK pathway [56]. Moreover, ERα signaling is recently clarified to preferentially polarize macrophages from a range of sources to an alternative phenotype [33]. Estrogen is able to inhibit NFκB-dependent inflammation by promoting synthesis of the negative regulator IκBα [57]. However, an opposed result shows that estrogen inhibits alternative activation polarization in a tumor-associated macrophage via the inhibition of JAK1-STAT6 pathway [58]. Although the role of estrogen in polarization of macrophages remains controversial, studies discussed above indicate the capacity of estrogen in mediating the recruitment of macrophages toward nerve fibers accompanied with M2 polarization of macrophages (Fig. 1).

### Neurogenesis is regulated by macrophages in an estrogen-dependent manner

Aberrant of innervation in endometriotic lesions is also demonstrated to be involved in the pathogenesis of endometriosis-associated pain [59]. Pain sensation of endometriosis is correlated with neural hypertrophy and increased neural density in the endometriotic lesion [60, 61]. Further studies discovered that there exists an imbalanced distribution of sensory, sympathetic and parasympathetic nerve fibers in endometriosis [7]. As a result, abnormal neurogenesis is important in endometriosis. Since the coexistence of macrophages and nerve fibers as well as their close correlation, macrophages can affect the neurogenesis in endometriosis. In addition, the secretion of multiple NGF from macrophages can be induced by estrogen, indicating a pivotal role of estrogen in mediating the above process [62].

#### Neurotrophins

Neurotrophins (NTs) belong to one of the trophic factor family. NTs play an important role in mediating the survival, growth and programmed cell death of nerves [63]. Members of the NTs increasingly expressed in endometriosis include NGF, brain-derived nerve growth factor (BDNF) and neurotrophin 3 (NT3) [64]. Previous studies reported that estrogen could increase NGF mRNA and NGF protein in murine uterus and salivary gland [65]. It has been demonstrated that macrophages are one of the important sources of NGF. 17β-estradiol can induce c-Fos expression and shift the composition of NGF promoter, AP-1 from c-Jun homodimers to c-Fos/c-Jun heterodimers in macrophages [62]. This transformation enhances the transcriptional activity of AP-1, leading to the increased expression of NGF in macrophages. In comparison with eutopic endometrium, estradiol also has the ability to promote the secretion of NT3 and BDNF in peritoneal endometriosis [13]. Moreover, estrogen can also up-regulate the expression of the receptor of NT3, tyrosine kinase receptor B (TrkB) and the receptor of BNDF, p75NTR on sympathetic neuron, which promote neuronal survival and sympathetic axonal growth in endometriotic milieu [66]. These evidence suggest a significant impact of estrogen on macrophages to potentiate neurogenesis in endometriosis via the dysregulation of neurotrophins.

#### Semaphorins

Semaphorins are a group of evolutionarily highly conserved surface or locally secreted nerve repellent factors that can regulate the axons migration, axonal growth and guidance [67]. Semaphorin 3A and its receptors (NRP1 and Plexin A1) have been clarified to decrease the innervation of sympathetic nerves in endometriosis [68]. Macrophages are reported to be one of the sources of semaphorin 3A [69]. Semaphorin 3F and 3C are also demonstrated to be released from macrophages in endometriosis [70]. Their receptors (NRP1 and NRP2) are positive in noradrenergic nerve fibers, which is associated with reduced sympathetic innervation [70]. As an estrogen-dependent disease, upregulation of estrogen is found to result in an increased expression of semaphorin 3F and lead to a sympathetic denervation in the rat uterus [71]. Some investigations further reveal a constant secretion of hepatocyte growth factor (HGF) from peritoneal macrophages in response to estrogen in endometriosis [72, 73]. Elevated levels of HGF from M2 macrophages can induce the expression and secretion of semaphorin 3A from myoblast [74]. It is reasonable to speculate the role of estrogen in regulating the secretion of semaphorins from macrophages, leading to the aberrant innervation in endometriosis.

#### Vascular epithelial growth factor

**Fig. 2:**
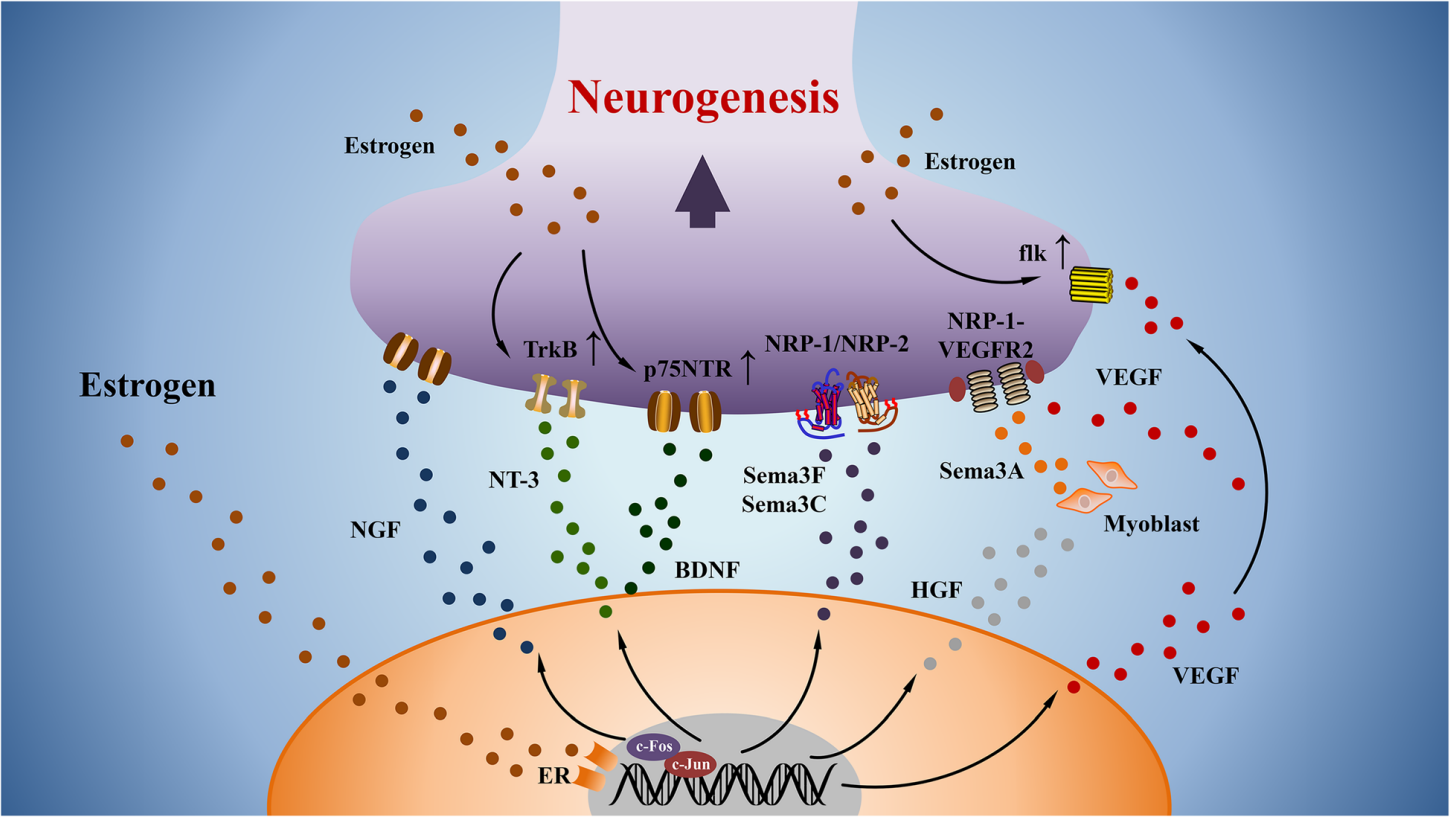
Neurogenesis regulated by macrophages in an estrogen-dependent manner. Estrogen can upregulate the expression of NGF from macrophages through the induction of c-Fos/c-Jun heterodimers. Expression of NT3 and BDNF is also increased in response to estrogen. Both of their receptors, TrkB and p75NTR, are upregulated under the effect of estrogen on peripheral nerves. The expression of Sema3F and Sema3C from macrophage can reduce the sympathetic innervation through binding their receptors, NRP1 and NRP2. High level of HGF from macrophage induces the expression of Sema3A from myoblast in response to estrogen. Estrogen can also stimulate the secretion of VEGF from macrophage and increase the expression of the VEGF receptor flk on peripheral nerve. Imbalanced concentration of VEGF and Sema3A can activate the nerve fiber via NRP1-VEGFR2 axis. Dysregulation of all nerve growth factors from macrophage and their receptors on nerve fibers eventually result in aberrant neurogenesis in endometriosis. NGF: nerve growth factor; NT3: neurotrophin 3; BDNF: brain-derived nerve growth factor; TrkB: tyrosine kinase receptor B; Sema3F, 3C, 3A: Semaphorin 3F, 3C, 3A; NRP1, 2: neuropilin1, 2; HGF: hepatocyte growth factor; VEGF: vascular epithelial growth factor; flk: vascular epithelial growth factor receptor 2

Besides stimulating vascular growth and increasing vascular permeability, vascular epithelial growth factor (VEGF) can also be a neurotrophic factor to stimulate axonal outgrowth. The receptor of VEGF, flk1, is up-regulated in peripheral ganglia in response to axonal injury [75]. High expression of flk1 have been confirmed to mediate the pathogenesis of endometriosis [76]. Further activation of flk1 results in the survival of nerve cells and stimulation of axonal outgrowth. In addition to acting locally at the level of the axons, VEGF has the ability to be transported retrogradely and acts at the level of the neuronal cell bodies [75]. Moreover, NRP1 is a co-receptor of VEGFR2. Elevated concentration of VEGF can activate VEGF-NRP1-VEGFR2 signaling, providing chemoattractive cues for sympathetic nerve outgrowth [77]. Intriguingly, activated macrophages are a major source of VEGF in endometriosis [78]. Most importantly, the secretion of VEGF from macrophages is regulated directly by estrogen. mRNAs encoding for estrogen and progesterone have been presented in peritoneal macrophages [72]. Macrophage-derived prostaglandin E2 elevates the level of estrogen in endometriosis, which subsequently activates macrophages to release VEGF [79]. Estradiol can also upregulate the expression of flk1 in uterus, mediating the progression of disease [80]. Therefore, in response to abnormal level of estrogen, macrophages are capable to release VEGF promoting aberrant neurogenesis in endometriotic environment (Fig. 2).

## The role of estrogen in inflammation and pain in endometriosis

Endometriosis is considered to be a chronic inflammatory disease and multiple molecules are demonstrated to contribute to this inflammatory milieu [81]. Since the neuroimmune communication has been identified in endometriosis, the inflammation resulting from the interplay between macrophage and nerve attracts more and more attention for the mechanism of endometriosis-associated pain [82]. According to the role of estrogen in nerve-macrophage interaction discussed above, dysregulation of estrogen exhibits a villainous role to modulate local inflammation in endometriosis and thus resulting in endometriosis-associated pain.

### Inflammation-induced peripheral nerve sensitization in an estrogen-dependent manner

Peripheral nerve sensitization is a process involving reduced threshold of ion channels, increased membrane excitability, and increased nociceptors expression. Inflammatory cytokines in endometriotic milieu can influence these aspects by binding to their corresponding nociceptors on peripheral nerves. Macrophages are thought to be the major source of inflammatory mediators, which can subsequently sensitize peripheral nerve fibers. The mRNA concentration of transient receptor potential vanilloid 1 (TRPV1) and transient receptor potential cation channel, subfamily A, member 1 (TRPA1) are significantly higher in women with endometriosis [83]. TNFα, IL1β and monocyte chemotactic protein 1 (MCP1) from macrophages can influence the membrane potential of sensory nerve via TRPV-1-dependent mechanism [83–85]. The NGF from macrophages can stimulate the activation of TRPV1 receptors through the activation of NGF receptors on neurons. The voltage-gated sodium channels, encoded by SCNA genes, are also stimulated by MCP1 through a CCR/Gβγ-dependent mechanism [86]. P2X (purinergic X) receptors, are another increased nociceptor in endometriosis which can be activated by extracellular ATP [87].As mentioned above, aberrant expression of ERα and ERβ in macrophages of endometriosis is correlated with the function of inflammatory cytokines, indicating the active role of estrogen in the secretion of inflammatory cytokines from macrophages [31]. The expression of those nociceptors discussed above is also up-regulated by estrogen in endometriotic cell-derived sensory neurons. These alternations by estrogen may be related to the presence of specificity protein 1 (SP1) transcription factor binding sites in the promoter regions of nociceptive genes [87]. In short, estrogen not only mediates the secretion of inflammatory cytokines from macrophages to activate nociceptors, but also increases the expression of nociceptors on peripheral nerves, which eventually exacerbates the local inflammation to induce peripheral hyper-sensitization.

### Neurogenic inflammation mediated by estrogen in endometriosis

**Fig. 3:**
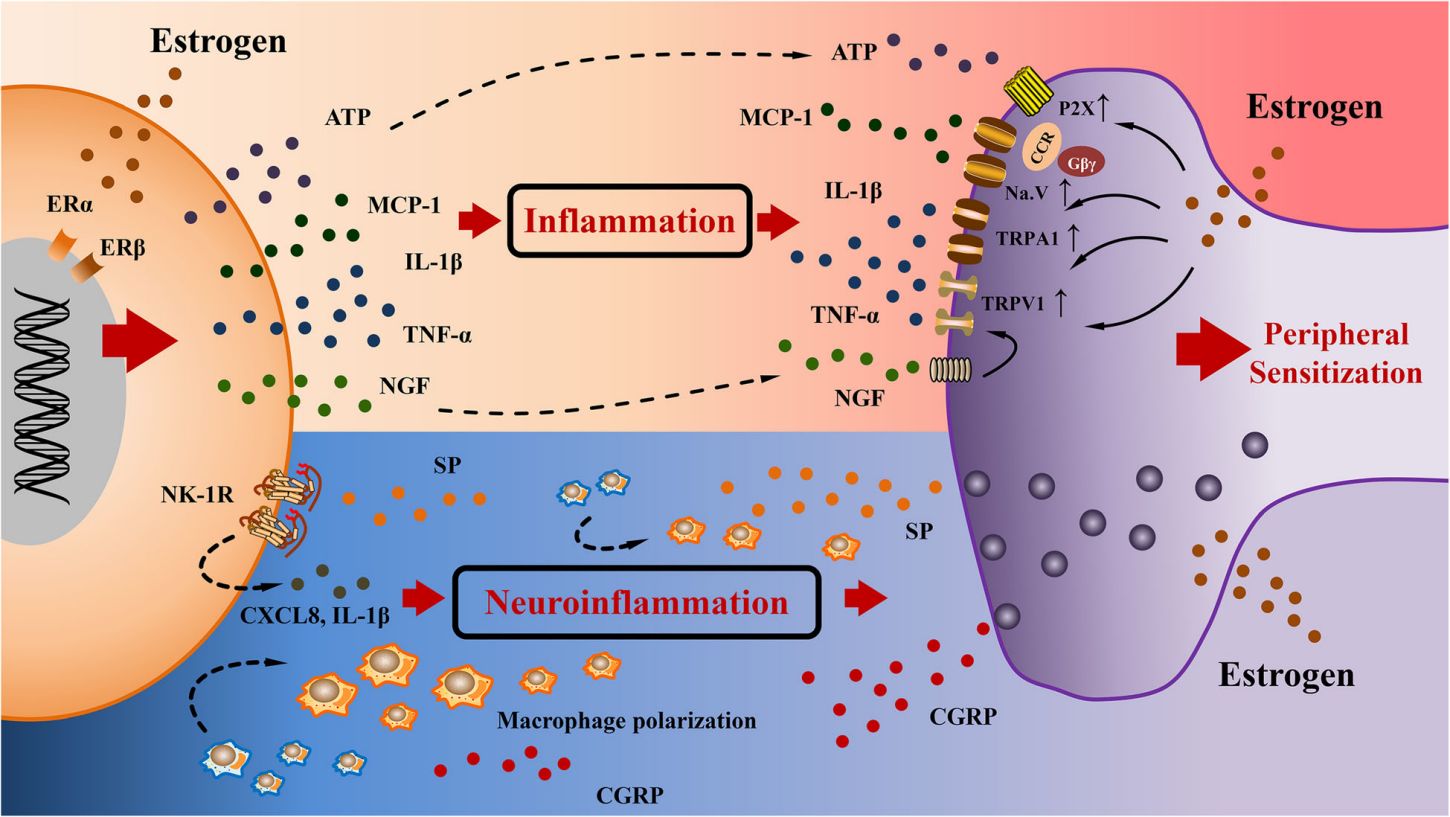
Role of estrogen in inflammation in endometriosis. Estrogen not only stimulates the secretion of inflammatory cytokines from macrophages, but also upregulate the expression of nociceptors on nerve fibers. IL1β, MCP1 and TNFα from macrophage can activate TRPA1 and TRPV1 on peripheral nerves. NGF from macrophages can also activate TRPV1, resulting in cross-sensitization of the TRPA1 and TRPV1 receptors. The interaction of MCP1 and its receptor Na.V sensitizes nerve fiber through a CCR/Gβγ-dependent mechanism. Macrophage-derived ATP stimulates the action of nerve fiber via P2X purinoreceptor. Persistent stimulation of peripheral nerve can promote the secretion of inflammatory neurotransmitters from nerve fibers. Expression of SP and CGRP can be modulated by estrogen. Release of SP from nerve fibers activates its receptor NK1R on macrophages in response to estrogen, resulting in up-regulation of CXCL8 and IL1β form macrophage. The activity of SP and CGRP can also alter the polarization of macrophage toward M2 phenotype. Macrophage-derived inflammation and neurogenic inflammation are both regulated by estrogen inducing a vicious circle to aggravate peripheral sensitization MCP1: monocyte chemoattractant protein 1; TNFα: tumor necrosis factor α; TRPA1: transient receptor potential cation channel, subfamily A, member 1; TRPV1: transient receptor potential vanilloid 1; SP: substance P; CGRP: calcitonin gene-related peptide; NK1R: neurokinin 1 receptor; CXCL8: chemokine (C-X-C motif) ligand 8.

Sustained peripheral sensitization by the inflammatory mediators from macrophages elicits the release of neuropeptides with inflammatory and nociceptive function, which induces a vicious circle to deteriorate the progression of endometriosis-associated pain. TRPV1-positive nerves can induce a neurogenic inflammation through the release of substance P (SP) and calcitonin gene-related peptide (CGRP) [88]. Further research demonstrates that estrogen can modulate corneal sensitivity by up-regulation of SP [89].The release of CGRP is also regulated by 17β-estradiol in the visceral pain sensitivity, implicating the role of estrogen in regulating of inflammatory neuropeptides [90]. The function of SP has been reported to stimulate the secretion of chemokine (C-X-C motif) ligand 8 (CXCL8) and IL-1β from macrophages, which exhibits a classically pro-inflammatory profile [91]. Furthermore, activation of neurokinin 1 receptor (NK1R), the receptor of SP, also depends upon estrogen in uterine [92]. However, activation of NK1R on macrophage has a special effect on M2 polarization through activation of P13K/Akt/mTOR signaling pathway, which may be beneficial for the repair of nerve injury [93]. The function of CGRP also exhibit compromised feature to promote M2 polarization of murine peritoneal macrophages via calmodulin, PKC and PKA pathway, leading to an attenuation of inflammation and tissue damage [94]. Although neuropeptides released from peripheral nerves generate a positive feedback to amplify the inflammation between nerves and macrophages, their function on macrophages polarization suggest that more evidence is necessary to clarify the effect of estrogen on these neuropeptides in endometriosis (Fig. 3).

## Implication of estrogen in regulating the interaction of nerves and macrophages in endometriosis

The role of estrogen in regulation of nerve-macrophage communication has been emphasized in the recruitment of macrophages, neurogenesis and peripheral sensitization. Targeting estrogen levels, the receptors on macrophages and nerve fibers may be a potential approach to prevent the progression of endometriosis. GnRH antagonists are popular drugs which down-regulates the release of follicle stimulating hormone (FSH) and luteinizing hormone (LH) by blocking the GnRH receptor in the pituitary cells, resulting in the suppression of ovulation [95]. Danazol is widely used and promotes endometrial atrophy by increasing free testosterone levels leading to a hypoestrogenic state [96]. A recent study developed two ER ligands, chloroindazole (CLI) and oxabicyloheptene sulfonate (OBHS), with CLI exhibiting ERβ-dependent activity and OBHS displaying ERα-preferential binding selectivity. Both ligands are optimized to have strong anti-inflammatory activity. OBHS and CLI can reduce innervation and neuron activation though down-regulation of COX2 and NGF [97]. The infiltration of macrophages is also completely prevented by the treatment with OBHS and CLI. Selective estrogen receptor modulators (SERMs) are drugs with both agonistic and antagonistic activity at the ER. It is providing new treatment strategies for endometriosis [98]. Administration of raloxifene is effective in the treatment of chronic pelvic pain in women with endometriosis [97]. The mechanism of raloxifene action relies on its anti-inflammatory properties, exemplified by a decrease in M1 monocytes, macrophage density, and the NFκB response associated with a decrease in NO, IL-1β and IL-6 production [99]. A study has shown that there is an earlier return of pelvic pain in raloxifene group compared to the placebo group, hindering further development of raloxifene [100]. For recurrence of chronic pain, accurate targeting of the ER in macrophages and nerve fibers has potential value in decreasing the occurrence of peripheral sensitization and progression of endometriosis.

## Conclusion

**Fig. 4:**
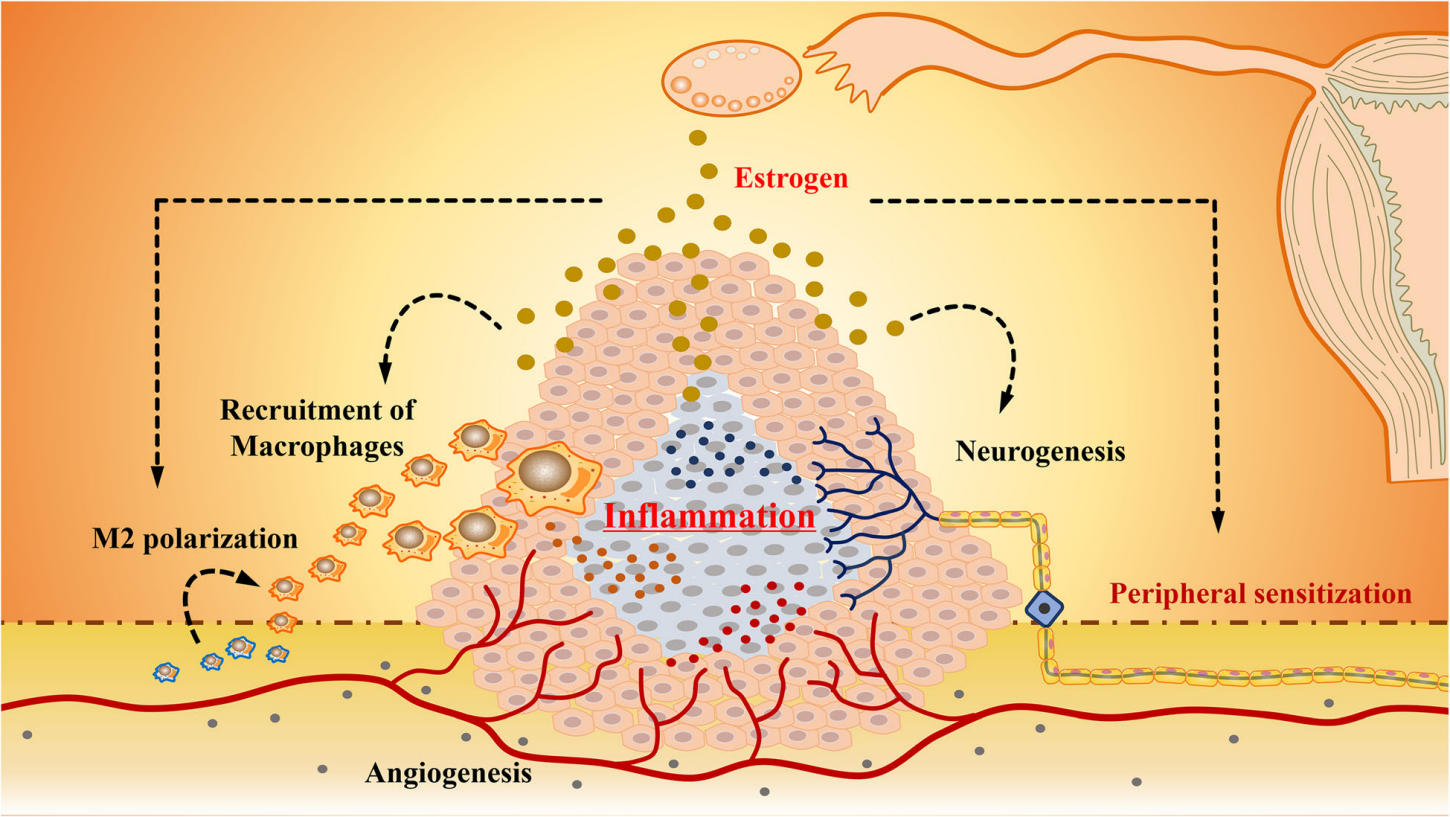
Significance of estrogen in endometriosis. Dysregulation of estrogen in endometriosis can mediate multiple aspects in endometriosis, including recruitment and polarization of macrophages, neurogenesis and angiogenesis. The interaction of macrophages and nerve fibers regulated by estrogen establish a vicious circle, aggravating inflammation in endometriotic milieu. Persistent inflammatory stimulation on peripheral nerve fiber result in peripheral sensitization, therefore exacerbating the progression of endometriosis

Dysregulation of estrogen in endometriotic milieu not only mediates the recruitment of macrophages in response to the chemokines from peripheral nerves, but also promotes neurogenesis within the microenvironment by acting on macrophages. The villainous communication between macrophages and nerve fibers has been demonstrated to be enhanced by the aberrant level of estrogen, providing a hypothesis in endometriosis-associated pain (Fig. 4). However, the role of estrogen and its receptors in the progression of endometriosis still require to be clarified. ER in macrophage polarization and different nerve fibers are still controversial. Details of the recruitment and polarization of macrophages in response to estrogen need to be further investigation. Although estrogen can alter the sensitization of peripheral nerves, evidence about the molecular mechanism of estrogen on the interaction of nerves and macrophages is not fully comprehensive. In conclusion, a better understanding of estrogen in the interaction of nerves and macrophages inspires a novel insight of endometriosis-associated pain and provides a new strategy for diagnosis and a potentially valuable target for the treatment of endometriosis-associated pain.
